# Differential Responses to Food Price Changes by Personal Characteristic: A Systematic Review of Experimental Studies

**DOI:** 10.1371/journal.pone.0130320

**Published:** 2015-07-07

**Authors:** Anja Mizdrak, Peter Scarborough, Wilma E. Waterlander, Mike Rayner

**Affiliations:** 1 British Heart Foundation Centre on Population Approaches for Non-Communicable Disease Prevention, Nuffield Department of Population Health, University of Oxford, Oxford, United Kingdom; 2 National Institute for Health Innovation, School of Population Health, University of Auckland, Auckland, New Zealand; University of Washington, UNITED STATES

## Abstract

**Background:**

Fiscal interventions to improve population diet have been recommended for consideration by many organisations including the World Health Organisation and the United Nations and policies such as sugar-sweetened beverage taxes have been implemented at national and sub-national levels. However, concerns have been raised with respect to the differential impact of fiscal interventions on population sub-groups and this remains a barrier to implementation.

**Objective:**

To examine how personal characteristics (such as socioeconomic status, sex, impulsivity, and income) moderate changes in purchases of targeted foods in response to food and beverage price changes in experimental settings.

**Design:**

Systematic review

**Data Sources:**

Online databases (PubMed, EMBASE, Web of Science, EconLit and PsycInfo), reference lists of previous reviews, and additional data from study authors.

**Study Selection:**

We included randomised controlled trials where food and beverage prices were manipulated and reported differential effects of the intervention on participant sub-groups defined according to personal characteristics.

**Data Analysis:**

Where possible, we extracted data to enable the calculation of price elasticities for the target foods by personal characteristic.

**Results:**

8 studies were included in the review. Across studies, the difference in price elasticity varied from 0.02 to 2.43 between groups within the same study. 11 out of the total of 18 comparisons of own-price elasticity estimates by personal characteristic differed by more than 0.2 between groups. Income related factors were the most commonly considered and there was an indication that own-price elasticity estimates do vary by income but the direction of this effect was not clear.

**Conclusion:**

Experimental studies provide an opportunity to examine the differential effects of fiscal measures to improve population diets. Patterns in price sensitivity by personal characteristics are complex. General conclusions pertaining to the effects of personal characteristics on price sensitivity are not supported by the evidence, which shows heterogeneity between studies and populations.

**Trial Registration:**

PROSPERO CRD42014009705

## Introduction

Fiscal interventions to improve diet are measures which induce changes in the price of foods and/or beverages, with the aim to impact on dietary patterns. These policies can differ in the dietary problem they set out to address, the products targeted by the intervention, and the magnitude and direction of price changes. Examples of fiscal interventions to improve diet include sugar-sweetened beverage taxes, fruit and vegetable subsidies, and snack food taxes [1].

There is a growing body of evidence suggesting that healthier dietary options tend to be more expensive than less healthy options [2] and lowering the price of healthier foods and raising the price of less healthy foods shifts purchases toward healthier options [3]. In recognition of their potential to change behaviour, fiscal interventions feature in the set of interventions recommended by the United Nations and the World Health Organization as a means to improve population diets [4,5]. Fiscal interventions to improve diet have also begun to be implemented in a variety of countries; examples include Hungary’s junk food tax, France’s sugar-sweetened beverage tax, and sugar-sweetened beverage taxes applied at state and city levels in the USA [6].

Fiscal interventions may affect different subgroups of the population in different ways. For example, low income purchasers may react differently to changes in food price than high income purchasers [7]. A particular concern is the potential regressive nature of taxes, meaning that those with low incomes experience a larger proportional increase in the price of foods than those higher incomes. This may result in differential health impacts of fiscal interventions that could reduce or exacerbate health inequalities. Thow et al comment that to date there have been various estimates of the regressiveness of fiscal interventions [8], and this is in line with the conclusions of reviews examining modelling studies [9], experimental studies [10] and natural experiments [11]. More needs to be understood about these potential differential health impacts in order to balance concerns with respect to the regressive economic nature of fiscal measures to improve population diets [1,11].

Experimental studies are one method to examine the effects of fiscal interventions and a method by which the differential effects on population groups can be explored. Experimental studies in this field involve subjecting groups of participants to a proposed fiscal intervention in a controlled setting and measuring difference in real or hypothetical purchases between groups that are exposed and not exposed to the tax. A merit of experimental studies compared to other study designs is that the controlled nature of experimental settings can help to disentangle the effect of the pricing intervention from confounders. For example, price elasticities for food groups observed naturally (using panel data on natural price fluctuations over time) are subject to non-measured influences on consumption patterns over time, such as upwards trends in meat consumption associated with increases in GDP [12]. Price changes in natural experiments may be a result of the market reacting to changes in demand, rather than the influence on demand that is the assumed relationship. In addition, experimental studies avoid the problems of extrapolation (typically of responses under small irregular fluctuations to a larger permanent price change) inherent in modelling studies.

Experimental studies can also inform the policy making process by providing much needed evidence with respect to the potential benefits (e.g. expected revenue gains) and harms (e.g. unintended purchasing shifts) of a suggested pricing intervention without the need for existing price elasticity data. This means that experimental studies may be especially informative when the proposed scope of the tax is not limited to traditional food groups for which price elasticity data are collected; for example, in the case of nutrient based taxes often considered by public health researchers.

Finally, experimental studies have the potential to determine differential effects of food price changes as they often collect individual level data and can therefore compare the responses of different groups, and examine the interactions between multiple personal characteristics.

Whilst experimental studies have their strengths, there limitations mean they cannot be used in isolation to determine the effects of pricing interventions. For example, the population recruited for an experimental study may not be representative of the population in which a possible pricing intervention in planned, the setting may not reflect the usual setting in which purchasing decisions are made, and therefore the purchase changes observed may not be those that would be observed in a real-life context. Experimental studies are typically of limited duration and therefore may not be able to provide evidence of the long-term purchasing shifts a price change could induce. Despite these and other limitations, their strengths offer the opportunity to use the evidence gathered in experimental research to complement that from other sources. To date, there has been no systematic review of experimental studies published in the literature that specifically addresses how the effects of fiscal interventions vary by subgroups defined by personal characteristics (e.g. income; BMI). Although Epstein and colleagues [10] conducted a targeted review of the experimental literature examining the extent to which price changes influenced purchases, the focus of the review was not on potential moderators of price sensitivity, and therefore numeric estimates of the effects of moderators were not presented. Therefore, this review set out to examine how personal characteristics moderate purchases in response to price changes, utilising price elasticity measures. Price elasticity is defined as change in demand for a unit change in price and is a common metric that can be used to examine differential effects across a heterogeneous selection of included studies. Recent systematic reviews of other study designs have addressed the issue of differential effects of food price changes and limited consistency in the results has lead review authors to conclude that the patterns based on personal characteristics are not consistent [8,9,11]. For example, Green et al note that there are differences in price elasticities by income which vary by food category [11] and Eyles et al find differences across studies with respect to the degree to which pricing policies are regressive [9]. We focus exclusively on experimental studies and add a synthesised, numeric interpretation of the available experimental evidence base to the conclusions drawn by existing reviews of other study designs.

### Primary research question

The primary research question we address is ‘How do personal characteristics (such as sex, socioeconomic status, income, and impulsivity) moderate changes in purchases of targeted foods in response to food/beverage price changes in experimental settings?’

In this context, we have defined personal characteristics as modifiable and un-modifiable participant features that may influence the response of individuals to a change in food and beverage prices. This includes factors such as age, sex, personality measurements (e.g. impulsivity as measured by the stop-signal reaction task), socio-economic status, income and education. We focus on personal characteristics rather than individual characteristics due to the expectation that included studies may look at households’ rather than individuals’ purchases. This means that studies which determined socio-economic status at the household level, rather than at the individual level, would not have been excluded from our analysis; i.e. we could include studies where the participant’s socio-economic status was defined on the basis of the occupation of their partner. Fiscal interventions may also incorporate non-pricing elements such as coupons, but here we concentrate exclusively on pricing elements of fiscal interventions as coupons may operate on other decision making mechanisms and behavioural frameworks [13,14].

### Secondary research questions

In addition to the primary research question, we also aimed to address the following secondary research questions.

- How do personal characteristics (see definition above) moderate changes in purchases of *non-targeted* foods in response to food/beverage price changes in experimental settings?
- How do the following factors influence differential responses to food/beverage pricing intervention by personal characteristic:
  ∘ Magnitude of the experimental price change (i.e. do larger price changes have an effect that is different to smaller price changes?)
  ∘ Scope of pricing target (i.e. do interventions that target a broad range of products have a different effect than interventions targeting a narrow range of products?)
  ∘ Direction of price change (i.e. do individuals react to price increases and decreases in an equivalent fashion or does the direction of the price change have an independent effect?)
  ∘ Information about price change (i.e. does the method by which individuals are informed about the price change result in different responses?)
- How will changes in food price affect the total price of the diet for different groups defined by socioeconomic or other personal characteristics?

## Methods

### Identification of relevant studies

We systematically reviewed the experimental literature according to a pre-defined protocol which was registered with the PROSPERO International Prospective Register of Systematic Reviews (registration no. CRD42014009705). The protocol is available on the PROSPERO website at http://www.crd.york.ac.uk/PROSPEROFILES/9705_PROTOCOL_20140414.pdf (accessed September 2014) and in S1 Protocol.

PubMed, EMBASE, Web of Science, PsychInfo, and EconLit were searched to identify potentially relevant studies using equivalent search strategies (see Table 1). A broad selection of food related search terms was chosen and the search strategy itself did not include any mention of personal characteristics as we wanted the search to pick up any experimental literature that could help answer the research question. The searches and title/abstract screening were initially conducted in March 2014 and then updated in August 2014 as we were aware that at least one relevant study had been published since the initial searches were carried out.

**Table 1:** PubMed search strategy.

The same search terms were used in all databases, with variations in notation made to ensure searches were equivalent.	
1	food OR foods OR snack OR snacks OR beverage* OR “soft drink” OR soda OR “carbonated drink”
2	fruit* OR vegetable* OR cereal* OR candy OR sweets OR confectionary OR chocolate* OR meat OR dairy
3	sugar OR sugars OR sugary OR “energy dense” OR “energy density” OR fat OR fats OR saturates OR “saturated fat” OR salt OR sodium OR fibre OR fiber
4	1 OR 2 OR 3
5	tax OR taxation OR taxes OR taxed OR subsidy OR subsidies OR price OR prices OR discount OR discounts
6	experiment OR experimental OR trial OR test OR supermarket* OR shop OR shops OR store OR stores OR controlled OR participant* OR intervention OR interventions OR random OR randomised OR randomized
7	4 AND 5 AND 6

All search hits were imported into an EndNote X7 library and titles and abstracts were screened for relevance. A 10% sample of the search hits was cross-checked for relevance by a second researcher. Full text articles were retrieved where the title and abstract indicated that the article was potentially relevant. In addition, the reference lists of included studies and three relevant recent reviews [1,10,15] were hand searched to identify additional articles of relevance.

An article was included if it was a controlled experimental study that reported results stratified by socioeconomic status or other personal characteristics of study participants, examined the effect of price change(s) on food or beverage purchases, collected individual level data to determine the effect (i.e. excluding single site studies such as vending or cafeteria studies where the outcome was total sales of targeted food), and that had either the price elasticity or changes in purchases or consumption of targeted or non-targeted foods as an outcome measure.

An article was excluded if it examined price changes of alcoholic drinks in isolation, was a review or commentary article where no original data were presented, did not test pricing interventions in isolation from other interventions (e.g. tailored nutrition education, coupon-based intervention), examined exclusively children’s (<18 years of age) purchases, was published prior to January 1980, or was not available in English.

### Data extraction and analysis

For numeric data, data reported in the included studies were converted to own price elasticity values for the target food. Accompanied 95% confidence intervals were obtained using either the reported confidence intervals for change in purchases or by conducting a Monte Carlo analysis using reported standard errors around the purchasing estimates. To conduct the Monte Carlo analysis, we set up an Excel sheet to randomly select values from within the distribution of baseline and intervention purchases; this enabled the creation of 10,000 possible price elasticities assuming independence between the baseline and intervention purchases (a conservative assumption). The mean and 95^th^ percentiles of these price elasticities gave the study price elasticity estimate and associated 95% confidence intervals, respectively. Alternatively, where changes in purchases of the target food were reported as number of calories from the target foods, price elasticity for a kilocalorie of the target food was calculated instead. In situations where an effect by a personal characteristic was reported but complete data were not given, study authors were contacted and asked to provide these data. We assumed that purchases in the control and intervention groups were normally distributed and independent of each other. This method means that the confidence intervals represent a conservative estimate and may be wider than the confidence intervals that would actually be observed. A meta-analysis was not undertaken as it was deemed inappropriate to combine price elasticities from differently defined personal characteristic groupings, countries, experimental settings, purchasing tasks and/or food groups. Studies were grouped according to the type of personal characteristics that were analysed: non-modifiable (age, sex), individual (personality (e.g. impulsivity, dietary restraint), BMI) and societal (income, socio-economic status, education). Where applicable, the results of fully adjusted models are the results reported in the data extraction table. Where relevant data were reported, cross-price elasticities for non-target foods were derived in the same way as own-price elasticities. A narrative summary of the available evidence on the secondary research questions is presented in the results section.

### Quality of included studies

The quality of included studies in relation to the primary research question was assessed according to the Cochrane Risk of Bias tool [16] and is tabulated in the results section. Nine questions across five domains of bias were used to assess study quality. Information from included studies was supplemented by information from study authors where answers were unclear from the manuscript; all study authors were contacted and additional information was obtained for six of the studies.

## Results

### Description of included studies

**Fig 1:**
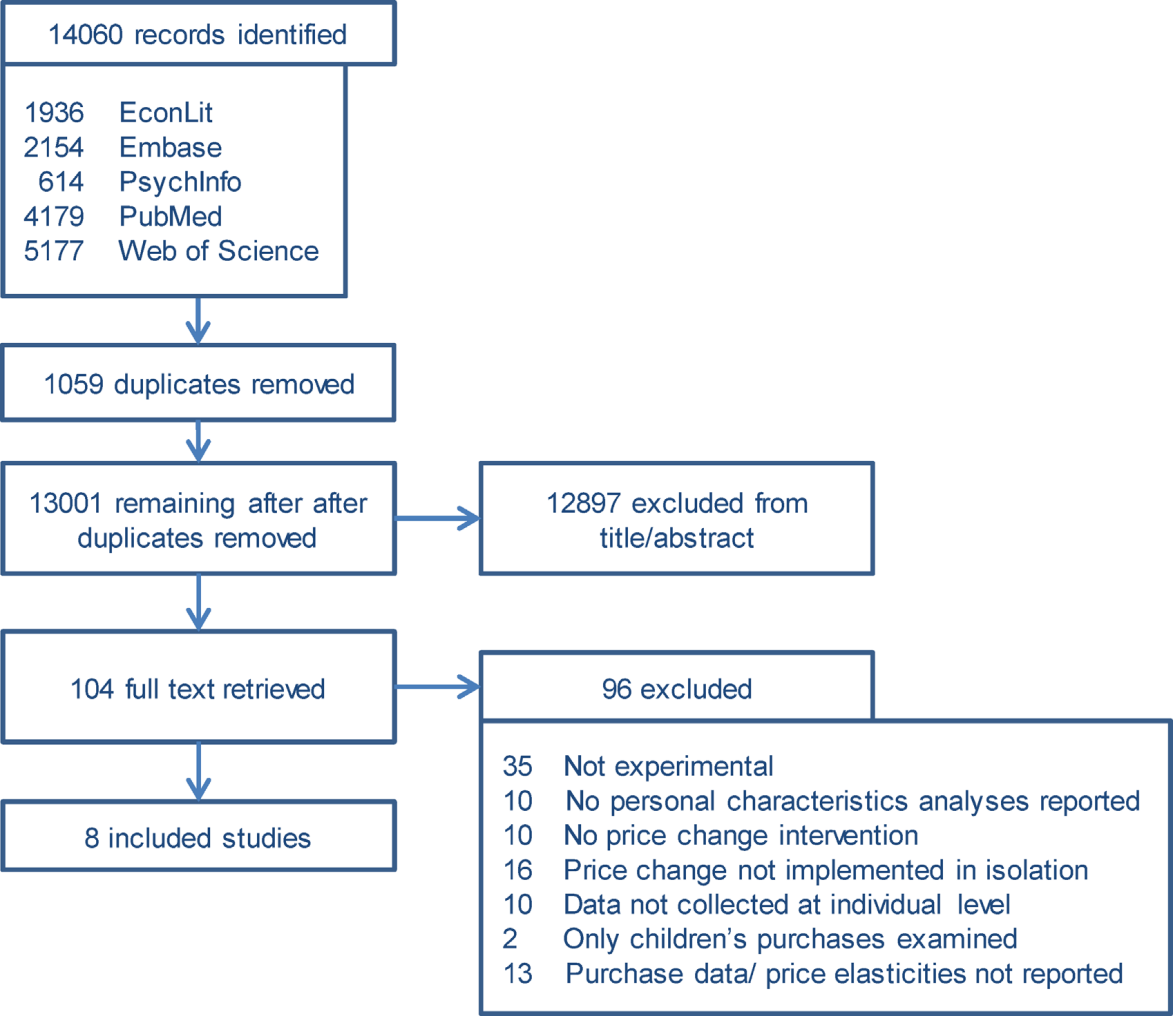
PRISMA flow diagram.

In total, 13,001 unique references were identified through the search strategy. From these, a total of eight articles were deemed eligible for inclusion in the present review. A PRISMA 2009 flow diagram is presented in Fig 1 with reasons for exclusion detailed. No additional studies were identified through hand searches of the reference lists of included articles or relevant reviews.

A summary of the included studies is given in Table 2. Six studies examined income [17–22], three examined BMI [18,19,22], two examined ethnicity/minority status [17,20]. Education [17], sleep deprivation [23], age [22], hunger [22], impulsivity [24], sex [20], price perception [20] and habit strength [20] were each examined in one of the included studies.

**Table 2:** Summary of included studies.

Author (Year)	Country	Setting	Design of study	Duration of study	Target of pricing intervention	Intervention conditions^a^	Personal characteristic(s) examined	Analysis type
Blakely (2011)	New Zealand	Leading supermarket chain	2x2 factorial randomised controlled trial	12wk baseline; 24wk intervention; 24wk follow-up	‘Healthier’ foods as assessed by a modified version of the New Zealand Heart Foundation Tick nutrient profile model	a) Control; b) Tailored nutrition education; c) 12% discount on ‘healthier foods’; d) Both tailored nutrition education and 12% discount on healthier foods	Ethnicity, Education, Household income	ANCOVA
Chapman (2013)	Sweden	Controlled laboratory setting	Three repeat purchasing tasks following a (randomised) night of either sleep or total sleep deprivation	Two laboratory visits (sleep and total sleep deprivation)	HED foods	Randomised to: i) Total sleep deprivation; ii) Sleep. Purchasing tasks were:1) Control; 2i) 25% price increase on HED^b^ foods; 2ii) 25% price decrease on HED^b^ foods	Sleep deprivation	ANOVA
Darmon (2014)	France	Computer shopping task conducted in the laboratory	Four repeat purchasing tasks	One laboratory visit	Fruit and vegetables and ‘other healthy products’ as defined by a nutrient profiling model (SAIN/LIM)	1) Software test; 2) Control; 3) 30% fruit and vegetable subsidy; 4) 30% price increase on unhealthy foods and 30% price decrease on healthy foods (including fruit and vegetables)	Income	General linear model
Epstein (2007)	USA	Controlled laboratory setting	Six repeat purchasing tasks following randomisation to price change target	Single laboratory visit lasting approximately two hours	Either HED or LED foods (randomly assigned)	Randomised to either: a) HED^b^ price changes; or b) LED^c^ price changes. This was followed by the following tasks (presented in random order): i) $15 per person budget, target 75% of reference price; ii) $15 per person budget, target at reference price; iii) $15 per person budget, target 125% of reference price; iv) $30 per person budget, target 75% of reference price; v) $30 per person budget, target at reference price; vi) $30 per person budget, target 125% of reference price	Study income, Obesity	Regression
Epstein (2010)	USA	Controlled laboratory setting	Five repeat purchasing tasks presented in a randomised, counterbalanced order	Single laboratory visit lasting approximately two hours	High and low CFN^d^ foods	i) Control; ii) Low CFN^d^ prices lowered 25%; iii) Low CFN^d^ prices lowered 12.5%; iv) High CFN^d^ prices raised 12.5%; v) High CFN^d^ prices raised 25%	Age, Minority status, BMI, Family income, Hunger	Regression
Giesen (2012)	Netherlands	Computer shopping task conducted in the laboratory	Three repeat purchasing tasks	Single laboratory visit	HED and LED foods	1) Control followed by 2i) HED^b^ price increase of 50%; and 2ii) LED^c^ price decrease of 50% (counterbalanced order)	Impulsivity (as measured by the stop-signal reaction time test)	ANOVA
Nederkoorn (2011)	Netherlands	Computer shopping task conducted remotely	Two condition randomised controlled trial	Single remote analogue purchasing task	HED foods	a) Control; orb) 50% tax on HED^b^ products	Budget, Weight status	Regression
Waterlander (2012)	Netherlands	Virtual Supermarket	Two condition randomised controlled trial	Single remote analogue purchasing task	Fruits and vegetables	a) Control; orb) 25% discount on fruit and vegetables	Sex, Budget, Price perception score, Habit strength	ANCOVA

^a^ Roman numerals (e.g. i, ii, iii) indicate participants were assigned to each condition in a randomised order. Alphabet characters indicate that participants were randomised to only one of the conditions presented. Numbers indicate where the order of conditions was pre-specified.

^b^ HED: High energy density

^c^ LED: Low energy density

^d^ CFN: Calorie for Nutrient

In one of the included studies, participants made purchases in a real-life supermarket [17], three studies utilised an experimental (laboratory) store [19,22,23], and four studies utilised computer based stores (three used an analogue online shop [18,21,24] and one used a virtual supermarket [20]). Three studies were based in the Netherlands [18,20,24], two in the USA [19,22], and one in New Zealand [17], France [21] and Sweden [23].

A price change was defined based on foods’ energy content in five of the studies [18,19,22–24], a nutrient profiling model in two studies [17,21] and fruits and vegetable subsidies were tested in two studies [20,21]. One of these studies [21] looked at both a fruit and vegetable subsidy and a nutrient profiling model based tax. Of the studies that had an energy based price change, four changed prices based on the number of calories per gram [18,19,23,24] and one changed prices based on the calorie for nutrient index [22]. The magnitude of the price changes examined ranged from 12.5% [17] to 50% [18,24]. The baseline/ control prices in all eight studies were based on actual prices of products in a comparable real world setting. Five studies examined a combination of price increases and decreases [19,21–24], two examined price decreases only [17,20], and one examined price increases only [18].

Although all included studies had an experimental design, there was considerable variation in study procedures, the primary outcome measure recorded by the researchers, and the analytic method used to present the results. Broadly, six studies included one experimental session/task [18–22,24] (often with multiple tasks in the single session [19,21,22,24]), one study required participants to visit the experimental laboratory on two occasions [23], and the final study tracked actual participant purchases over 50 weeks (12 baseline, 24 intervention, 24 follow-up) [17]. Sample size ranged from 16 [23] to 1104 [17]; four of the studies had sample sizes less than 100.

### Primary outcome: price elasticities for target foods

For six studies, it was possible to convert reported results to point price elasticities for the target food, by personal characteristics. Overall, it was possible to calculate 18 sets of own-price elasticity estimates (i.e. calculations of price elasticity by personal characteristic); these are displayed in Table 3.

**Table 3:** Own-price elasticities for target products by participant sub-groups.

	Personal characteristic	Personal characteristic measure	Author name (year)	Target food	Price change applied to target food ^a^	Personal characteristic categories	Price elasticity result (95% confidence intervals)	Difference between groups? ^c^
Non-modifiable	Impulsivity	Stop signal reaction time (SSRT) test score	Giesen (2012)	High energy density foods	+25%	Low SSRT score	0.41(-0.46, 2.08)	No
						High SSRT score	0.49 (-0.68, 1.21)	
				Low energy density foods	-25%	Low SSRT score	-1.45 (0.41, 3.18)	Yes
						High SSRT score	0.00 (-1.06, 0.64)	
	Ethnicity	Self-report ethnicity response	Blakely (2011)	‘Healthier foods’ as defined by a modified version of the New Zealand Heart Foundation Tick nutrient profile model	-12.50%	Maori	0.24 (1.75, -1.27)	Yes
						Pacific	-2.19 (-0.11, -4.26)	
						European/other	-0.99 (-0.58, -1.38)	
Modifiable	Obesity	BMI (binary)	Epstein (2007)	High energy density foods	+/-25%	Non-obese	-1.05 (-1.24, -0.86)	Yes
						Obese	-0.77 (-0.99, -0.55)	
				Low energy density foods	+/-25%	Non-obese	-0.76 (-0.87, -0.64)	No
						Obese	-0.83 (-0.99, -0.55)	
			Nederkoorn (2010)	High energy density foods (defined as >300kcal/100g)	+50%	Lean	-0.43 (-1.33, 1.07)	No
						Overweight	-0.34 (-1.01, 0.71)	
	Sleep deprivation	Binary	Chapman (2013)	High calorie foods	-25%	Total sleep deprivation	-0.94 (-2.14, -0.02)	No
						Sleep	-0.77 (-1.93, 0.12)	
					+25%	Total sleep deprivation	-0.98 (-1.81, 0.01)	Yes
						Sleep	-1.19 (-1.73, -0.51)	
Societal	Income	Study income (per household member)	Epstein (2007)	High energy density foods	+25%	US $15	-1.80 (-2.02, -1.57)	Yes
						US $30	-1.17 (-1.44, -0.89)	
					-25%	US $15	-1.00 (-1.66, -0.37)	Yes
						US $30	-1.42 (-1.91, -0.96)	
				Low energy density foods	+25%	US $15	-0.82 (-0.90, -0.75)	No
						US $30	-0.80 (-0.91, -0.69)	
					-25%	US $15	-1.18 (-1.50, -0.85)	No
						US $30	-1.11 (-1.36, -0.86)	
		Daily household grocery budget	Nederkoorn (2010)	High energy density foods (defined as >300kcal/100g)	+50%	<10€	-0.44 (-1.33, 1.07)^b^	Yes
						10–20€	-0.46 (-0.87, 0.03)^b^	
						>20€	-0.26 (-0.71, 0.32)^b^	
		Household income	Blakely (2011) **	‘Healthier foods’ as defined by a modified version of the New Zealand Heart Foundation Tick nutrient profile model	-12.50%	<NZ $60,000	-0.19 (0.22, -0.60)	Yes
						>NZ $60,000	-0.06 (0.23, -0.34)	
						Declined to answer	-1.28 (0.63, -3.19)	
		Household income	Darmon (2014)	Fruit and vegetables	-30%	Low income	-0.82 (-0.70, -0.94)	Yes
						Medium income	-1.28 (-1.08, -1.47)	
				Healthy products (including fruit and vegetables)	-30%	Low income	-0.40 (-0.39, -0.41)†	Yes
						Medium income	-0.71 (-0.66, -0.76)†	
				Unhealthy products	+30%	Low income	-0.75 (-1.28, -0.22)†	Yes
						Medium income	-1.18 (-0.87, -0.48)†	
	Education	Highest qualification obtained	Blakely (2011) **	‘Healthier foods’ as defined by a modified version of the New Zealand Heart Foundation Tick nutrient profile model	-12.50%	Nil/Secondary	-0.94 (-0.36, -1.51)	No
						Tertiary/trade/other	-0.78 (-0.22, -1.35)	

^a^ Given for reference purposes price elasticities given are point price elasticities and the calculations are therefore partially dependent on the magnitude of the price change

^b^ Value reported is the price elasticity for a calorie of the targeted food

^c^ ‘Yes’ values indicate that the difference between price elasticity values of the pairwise groups was greater than +/-0.2.

^†^ These calculations ignore the impact of the price changes of the non-target food and are therefore likely to be an overestimation of the true own price elasticity as the price increase on unhealthy products and price decrease on healthy products was applied simultaneously

** Data not published in included manuscript, provided by Tony Blakely and Yannan Jiang (personal communication)

Only two studies [23,24] provided enough data to calculate price elasticities for all the personal characteristics examined. Four studies provided partial data and the authors were contacted for further details [17–19,21]. Two studies [20,22] only reported that there were no significant interactions with modifiers/interaction terms. The confidence intervals for price elasticity estimates in two studies [17,21] could be obtained using the confidence intervals for the change in purchases between the baseline and intervention phases. For the remaining studies, we conducted a Monte Carlo analysis to obtain confidence intervals around the price elasticity estimates.

The own-price elasticities calculated span six personal characteristics: impulsivity, ethnicity, obesity, sleep deprivation, income, and education. Apart from for income and obesity, the price elasticities calculated for a personal characteristic relate to the findings from a single study. A difference of greater than 0.2 in the mean point price elasticity estimate between groups was chosen as representing a noteworthy difference between groups. A value of 0.2 represents a difference in price elasticities that is greater than differences between population subgroups that have been observed under natural price fluctuations, as reported in a recent systematic review [11]. Eleven out of 18 own price elasticity estimates differed by more than 0.2 between personal characteristic groupings. The greatest difference in own price elasticities between groups was observed between ethnic groups in the SHOP study (a difference in price elasticity of 2.43 between Maori and Pacific participants) [17].

We found a consistent indication of differences in own price elasticity estimates across all four studies which examined the effect of income [17–19,21]. However, there was no consistent pattern with respect to whether high income or low income groups were more price sensitive. Within studies that examined price changes across different foods, differences in price elasticity estimates by personal characteristic were often not concordant (i.e. large differences in price elasticity by personal characteristic for one set of target foods did not necessarily mean that there were large differences in the price elasticities in other target foods examined).

There were two sets of price elasticity estimates derived to represent the response to a price change in high energy dense foods by obesity status. Despite the scope of the tax being comparable across the two studies [18,19] the studies had contrasting findings; there was not a large difference in the Nederkoorn study [18] but there was in the Epstein (2007) [19] study. This may suggest that the impact of a particular personal characteristic may be context, or culturally specific.

For the personal characteristics where price elasticity estimates were derived from a single study, there is mixed evidence of an effect of personal characteristics. While there are differences of greater than 0.2 in the price elasticity for healthy foods based on ethnicity in the SHOP study [17], there is a considerable degree of overlap in the confidence intervals. In the same study, no considerable difference is observed by education group. For impulsivity and sleep deprivation, it appears as though there may be differential effects under some conditions but not others. In the impulsivity study [24], there is a difference of greater than 0.2 for low energy density foods but not for high energy density foods. In the sleep deprivation study [23], there is a difference of greater than 0.2 for a price increase but not for a price decrease in the target foods. This indicates that the effect of personal characteristics in responses to price changes may be complex, even within a single personal characteristic.

### Secondary research questions

#### How do personal characteristics affect changes in purchases of non-targeted foods in response to food/beverage price changes in experimental settings?

The differential effects of the applied price change on purchases of products other than the targeted product (e.g., cross price elasticity) was discussed in five of the included studies [19–24], with sufficient numeric data provided to estimate cross price elasticities by personal characteristic in four [19,21,23,24]. It was possible to obtain a total of 15 cross-price elasticity sets across four personal characteristics (impulsivity, income, sleep deprivation, and obesity); these are shown in Table 4. Six of the 15 cross price elasticity estimate sets had a difference of greater than 0.2 between groups. The greatest difference in cross-price elasticity was observed in the Giesen [24] study where there was a 0.57 difference in the cross-price elasticity of calories from HED foods when the price of LED foods was changed. Overall, there was no clear pattern of the effects of any personal characteristics on cross price elasticities. Income was the only personal characteristic for which there was data from more than one study. None of the income related cross-price elasticity differences represented a difference of more than 0.2. For impulsivity and obesity, some cross price elasticities showed a difference of greater than 0.2 while others did not. There were differences greater than 0.2 in the cross price elasticities by sleep deprivation status.

**Table 4:** Cross-price elasticities for non-target products by personal characteristic groups.

Personal characteristic	Personal characteristic measure	Author name (year)	Target food	Price change applied to target food(s)	Non-target product considered	Personal characteristic categories	Price elasticity result (95% confidence intervals)	Difference between groups?
Impulsivity	Stop signal reaction time	Giesen (2012)	High energy density foods	+25%	Kcal from medium energy density foods	Low SSRT score	0.15 (-0.53, 1.04)	No
						High SSRT score	0.07 (-0.74, 0.61)	
					Kcal from low energy density foods	Low SSRT score	-0.62 (-1.00, -0.07)	Yes
						High SSRT score	-0.05 (-1.01, 0.72)	
			Low energy density foods	-25%	Kcal from high energy density foods	Low SSRT score	-0.29 (-0.88, 0.57)	Yes
						High SSRT score	-0.82 (-1.81, -0.12)	
					Kcal from medium energy density foods	Low SSRT score	0.32 (-0.35, 1.19)	Yes
						High SSRT score	-0.32 (-1.17, 0.31)	
Income	Study income	Epstein (2007)	High energy density foods	+25%	Low energy density foods	US $15	0.29 (0.02, 0.58)	No
						US $30	0.17 (0.08, 0.43)	
				-25%	Low energy density foods	US $15	0.07 (-0.14, 0.28)	No
						US $30	0.04 (-0.14, 0.22)	
			Low energy density foods	+25%	High energy density foods	US $15	0.19 (-0.07, 0.48)	No
						US $30	0.16 (-0.02, 0.34)	
				-25%	High energy density foods	US $15	-0.57 (-1.01, -0.14)	No
						US $30	-0.40 (-0.66, -0.14)	
Income	Household income	Darmon (2014)	Fruit and vegetables	-30%	Other healthy products	Low income	0.03 (0.07, -0.01)	No
						Medium income	-0.16 (-0.14, -0.18)	
					Neutral products	Low income	0.26 (0.19, 0.34)	No
						Medium income	0.27 (0.23, 0.31)	
					Unhealthy products	Low income	0.14 (-0.44, 0.72)	No
						Medium income	0.08 (-0.57, 0.73)	
Sleep deprivation	Binary	Chapman (2013)	High calorie food items	-25%	Low calorie food items	TSD	-0.07 (-1.70, 1.16)	Yes
						Sleep	-0.37 (-1.92, 0.78)	
				+25%	Low calorie food items	TSD	0.37 (-1.05, 2.23)	Yes
						Sleep	0.93 (-0.45, 2.76)	
Obesity	Binary	Epstein (2007)	High energy density foods	+/-25%	Low energy density foods	Non-obese	0.22 (0.09, 0.34)	Yes
						Obese	-0.02 (-0.19, 0.15)	
			Low energy density foods	+/-25%	High energy density foods	Non-obese	-0.07 (-0.25, 0.10)	No
						Obese	-0.12 (-0.32, 0.08)	

#### Impact of magnitude, target, direction, and information on differential responses to food/beverage pricing interventions by personal characteristic

Two studies examined multiple price conditions for the target product(s) and three personal characteristics were considered in these studies (BMI, study income, and sleep deprivation) [19,23]. From this limited evidence base, it is not possible to draw conclusions with respect to how the direction of a price change might differentially impact different groups. There was no data from the included studies as to how the magnitude of price changes may impact differential effects (i.e. whether a price increase of 10% and 20% induce proportional purchasing changes) and the heterogeneity of studies even further prohibited such conclusions.

Four of the included studies [19,21,22,24] tested price changes on more than one set of target products. Three of these studies provided data that enabled the calculation of own price elasticities for the different target foods [19,21,24]. Within studies, price elasticity differences between personal characteristic groups did appear to differ based on the target of the price change. For example, there was not a considerable difference in the price elasticity estimates between low and high study income for low energy density foods but there was a difference in the price elasticity of high energy density foods in the Epstein (2007) study [19].

There was no evidence available from the included studies to enable us to examine the impact of information provision on differential impacts of price changes by personal characteristic. Although three studies [17,19,22] informed participants of the price changes that were implemented, none had an uninformed or differently informed comparison group.

#### How do changes in food price affect the total price of the diet for different groups defined by socioeconomic or other personal characteristics?

There was only one study that explicitly examined the impact of price changes on the total price of the diet [21]. The study tested two interventions (fruit and vegetable subsidy, and nutrient profile based changes); both price changes tested resulted in a reduction in daily expenditure. For the fruit and vegetable subsidy, low income participants reduced expenditure by a greater absolute amount than the median income participants. For the nutrient profile condition, the same absolute reduction in price was observed for both groups. The authors also reported the redistributive effects of the intervention and found that they were more favourable for the median income group than the low income group. Nederkoorn et al [18] discussed changes in total price of the diet but no differential effects by personal characteristic were noted.

### Study quality

Table 5 shows the judgements made in relation to the quality of included studies. No study was judged to be at low risk of bias across all domains, and there was considerable variance in the risk of bias across the five domains. There were no studies where the researchers were blinded to the allocation of participants, but there was often no or little contact with researchers post-randomisation and therefore the impact on results is likely to be minimal. Only two studies required participants to make purchases using their own money and therefore there is considerable risk of performance bias across the included studies.

**Table 5:** Assessment of the quality of included studies.

		Blakely (2011)	Chapman (2013)	Darmon (2014)	Epstein (2007)	Epstein (2010)	Giesen (2012)	Nederkoorn (2011)	Waterlander (2012)
Selection bias	Were participants randomised to the study [price] conditions?	Yes	Yes	No	Yes	Yes	Yes	Yes	Yes
	Were participant recruitment methods independent of personal characteristics?	No	Yes	No	Yes	Yes	Yes	Yes	Yes
Performance bias	Were participants blinded to the aims of the research study? i.e. blinded to the fact that the study changed prices	No	No*	No	No	No	Yes*	Yes	Yes
	Did the study design require participants to make actual purchases using their own money?	Yes	No	Yes	No	No	No	No	No
Detection bias	Were participants blinded to the outcome of interest? I.e. were participants aware of why prices changed	No*	Yes*	Yes	Unknown	Unknown	Yes*	Yes	Yes
	Were researchers blinded to the allocation of participants?	No*	No*	No	No	No	No*	No*	No
Attrition bias	Was complete outcome data obtained?	No	No	No	Yes	Yes	No	No	No
	Was attrition unrelated to the personal characteristics examined?	No	Yes	Unknown*	Yes	Yes	Yes*	Unknown*	Unknown*
Reporting bias	Did the study set out to look at differences by personal characteristics?	Yes	Yes	Yes	Unknown	Unknown	Yes	Yes	No

‘Yes’ responses represent low risk of bias; ‘No’ responses represent higher risk of bias.

*Indicates where study authors provided additional information to help clarify responses

## Discussion

This systematic review finds that price changes may have a differential effect amongst depending on purchasers’ personal characteristics. However, the results included in this review were typically under-powered as evidenced by the wide price elasticity confidence intervals. Therefore, it is possible that the patterns observed represent chance findings, especially in instances where a personal characteristic was only examined in a single study.

Differences in own price elasticities for different targets within studies suggest that the choice of target products may influence the extent to which responses vary by personal characteristic. This also appears to be the case in relation to cross price elasticities. However, owing to the limited data it is too early to draw conclusions with respect to either the personal characteristics or target foods for which this is relevant.

Observational studies suggest that there are differential effects by personal characteristics such as income [11] and we therefore need adequately powered experimental studies to test whether these effects are due to confounding. An adequate examination of the influences of personal characteristic on responses to food price changes is required if to assess whether existing dietary inequalities might be affected with the introduction of fiscal measures to improve population diets.

### Limitations of included studies

This systematic review identified a number of weaknesses in experimental studies conducted to date that examine the differential effect of food price changes by personal characteristic. Primarily, included studies did not have a large enough sample sizes to enable statistically significant differences by personal characteristics to be observed. We observed that the confidence intervals around own price elasticity estimates were often very wide which suggests that the studies were not adequately powered to examine the differential effects of the price changes implemented.

In several cases, sample sizes were not even sufficient to conclusively determine whether a price change resulted in increases or decreases in the purchase of target products in a particular sub-group of participants [17,18,23,24]. This may be partly due to the difficulty of recruiting from particular population sub-groups (e.g. [25]).

Another weakness of included studies was that continuously distributed personal characteristics were dichotomised, and the range in the personal characteristic measure was generally not reported, thus making it difficult to establish the homogeneity of the study sample. Studies where there is little difference in the personal characteristic measure across dichotomised groups may fail to identify differential effects due to the two groups being too similar to establish an effect. The extent to which this “false dichotomisation” may have influenced the power of studies to detect differential effects by personal characteristics is unclear.

### Limitations of the systematic review

The broad search strategy adopted for this review meant we were able to identify studies spanning a wide range of personal characteristics. Despite this, only eight relevant studies were identified. These were highly heterogeneous in the targeted foods, range of products available, price intervention applied, study task and personal characteristic measures used. The small number of heterogeneous studies meant that there were too few studies on specific personal characteristics and pricing interventions for general conclusions to be drawn. The variation in the study setting, price change interventions, shopping tasks conducted, and other factors also meant that it is not appropriate to conduct a meta-analysis and therefore pooled estimates of the effects of particular personal characteristics are not available.

The conversion of the results reported in included studies to a common metric enabled transparent comparison of results across studies. In some cases, price elasticities were obtained for calories from the target product rather than price elasticities for the amount of the target products purchased. Although price elasticities for calories and amount of product are not directly equivalent and therefore cannot be compared directly, both give an indication of the magnitude of a purchasing shift under a given pricing condition. A weakness of the approach of converting reported study results to price elasticities was that the confidence intervals were probably overestimates and this may partly account for the difficulty in exactly establishing differential effects by personal characteristic across the included studies.

Due to the lack of power of included studies to establish the statistical significance of differences between groups, we examined differences in the mean price elasticity estimates. A difference in price elasticities of greater than 0.2 was chosen to indicate that there were potentially differential effects according to personal characteristic, but this cut-off was somewhat arbitrary. In small studies with little power, price elasticities greater than 0.2 may occur simply by chance. In addition, very small differences in price elasticity across commonly consumed product categories may have a greater impact on diet than large differences in price elasticity for foods that are less commonly consumed. However, this is unlikely to have biased the findings of this review as all the included studies tested the effects of price changes across relatively broad food categories.

The secondary research questions that were included ensured that differential effects of a price change could be examined in the broadest sense possible. However, the small number of studies prevented being able to draw conclusions about differential effects of pricing interventions on substitution patterns, total price of the diet, and non-price elements of pricing interventions (e.g. information).

### Comparison with previous reviews

This review set out to systematically assess differential effects of food price changes and quantify these effects across included studies by using a common metric. This differs from Epstein and colleagues’ [10] targeted review of experimental studies which provided a broader narrative description of the experimental evidence base. Overall, they conclude that additional focused research is needed to better inform policy and state that there is a suggestion that some individual characteristics do moderate the effects of pricing interventions [10]. However, the authors do not quantify these effects as this was not the primary focus of their review. By adopting a systematic approach, we identified all the focused research that has been conducted and have ensured an unbiased representation of the totality of findings from the existing evidence base. The secondary research questions chosen in this review built partly upon Epstein and colleagues’ [10] earlier observations as we were intrigued as to what a systematic synthesis would find, and whether the seemingly growing popularity of the subject would yield novel findings from more recent literature. There were fewer studies included in this review due to the more focused nature of the research question and stricter inclusion criteria. For example, no vending machine studies were deemed eligible for this review as these studies did not collect data at the individual level.

Overall, reviews of fiscal interventions to improve population diets note the importance of considering the differential effects of fiscal interventions to improve population diets, and the limited evidence base that is currently available to address this issue [3,9,10]. Where differential effects have been observed, the focus has primarily been on differential effects for different socioeconomic groups, and the findings have tended to suggest that pricing strategies result in improved health benefits for lower socioeconomic groups, but that policies tend to be economically regressive [9]. From the experimental literature reviewed here, income related effects do appear to be present but the direction of these effects is unclear. In addition, this review observes that there may be other personal characteristics, such as ethnicity and obesity status, for which the differential effects may also be important to consider in order to avoid increasing health inequalities.

### Suggestions for future research

The findings of this systematic review indicate that future research should make a greater effort to test the differential effects of policy applicable price changes across population relevant sub-groups by recruiting sufficient sample sizes from the different subgroups for adequately powered statistical analyses. Explicit efforts should be made to establish the effects of pricing interventions on both own and cross price elasticity values and the overall diet by conducting experiments in realistic food purchasing settings with diverse food options such as supermarkets.

## Conclusions

Insight into the differential effects of fiscal interventions to improve population diets is important in order to establish the ultimate effects on food purchasing patterns, public health outcomes, and health disparities. Experimental studies provide an opportunity to examine the differential effects of fiscal interventions in detail. We find that there is some evidence of fiscal interventions having a differential impact depending on the personal characteristic of study participants, however the extent and nature of these differences remains unclear.

The limited sample sizes and heterogeneity of studies mean it is not possible to draw coherent conclusions with respect to specific personal characteristics or with respect to the exact magnitude or direction of differences. We recommend that future experimental studies ensure that they are adequately powered to examine relevant differential effects for the fiscal intervention that is being trialled, as there is an insufficient evidence base to date to be able to draw generalizable conclusions about the impact of particular personal characteristics.

## Supporting Information

S1 PRISMA Checklist 2009 Checklist. (DOC)

S1 Protocol Submitted to PROSPERO. (PDF)
