# Supplementary material for: Differential Responses to Food Price Changes by Personal Characteristic: A Systematic Review of Experimental Studies
Source: PLoS One. 2015 Jul 7;10(7):e0130320. doi: 10.1371/journal.pone.0130320 (PMC4494840; doi:10.1371/journal.pone.0130320)
Supplement: S1 Protocol — (PDF) [file pone.0130320.s002.pdf]

PROTOCOL

Differential responses to food price changes by personal characteristic: a systematic review of experimental studies

Diet related risk factors contribute significantly to the burden of disease. In high income countries, the combined influence of dietary factors and physical inactivity are attributable for over a quarter of deaths [1]. Fiscal measures have been proposed as one way of influencing consumers to make healthier choices as there is a growing body of research suggesting that lowering the price of healthier foods and raising the price of less healthy foods shifts purchases toward healthier options [2] and that healthier dietary options currently tend to be more expensive than less healthy options[3]. Fiscal interventions aimed at improving diets can differ in the dietary problem they set out to address, products targeted by the intervention, and the magnitude and direction of price changes. Although fiscal interventions may incorporate non-pricing elements such as labelling, coupons or advertising restrictions, here we concentrate exclusively to pricing elements of fiscal interventions as there is at least some evidence to suggest that non-pricing elements operate on other decision making mechanisms and behavioural frameworks[(e.g. 4,5)].

Before introducing food-related fiscal measures, it is important to gain in-depth evidence on the effectiveness of the measures with regard to the food purchases, and eventually population diets. In particular, it may also be important to assess how fiscal measures may affect those with particularly poor diets (regardless of income) or other personal characteristics to ensure that fiscal measures do not introduce additional or exacerbate existing health inequalities. Concerns with respect to the regressive nature of fiscal measures to improve population diets have been raised[6,7] and the effectiveness of a fiscal policy will necessarily rely on the extent to which fiscal measures are able to prevent or mitigate detrimental effects.

Experimental studies provide an effective way of examining the differential effects of fiscal policies on population sub-groups for a number of reasons. Experimental studies are able to expose study participants to the precise food pricing conditions that may come about as a result of fiscal measures to improve population diets and are less subject to the problems of extrapolating that are inherent in other methods of estimating the effects of fiscal policies. Experimental studies can collect individual level data and therefore compare the responses of different groups, and examine interactions between multiple personal characteristics (e.g. the interaction between budget and weight status in dietary choices[8]). Finally, the controlled nature of experimental settings can help to disentangle the effect of the pricing intervention from exogenous factors such as the accessibility of foods which may confound traditional price elasticity estimates.

Recent reviews of the effects of fiscal policies to improve population diets[2,7,9] highlight the need to gain a greater understanding of socioeconomic and other differential effects in responses to fiscal measures. To date, there has not been a review that specifically addresses how personal characteristics may influence responses to fiscal measures to improve population diets. This systematic review sets out to address this gap by examining the experimental literature. The

research questions that form the focus of this review, and the methods by which this systematic review will be conducted are outlined below.

Primary research question

- How do personal characteristics[a] (such as socioeconomic status, gender, impulsivity, income) moderate changes in purchases of targeted foods in response to food/beverage price changes in experimental settings?

Secondary research question

- How do personal characteristics moderate changes in purchases of non-targeted foods in response to food/beverage price changes in experimental settings?
- How do the following factors influence differential responses to food/beverage pricing intervention by personal characteristic:
    - Magnitude (i.e. do larger taxes have an effect that is greater than the absolute difference in price compared to smaller taxes?)
    - Target (i.e. do interventions that target a broad range of products result in a greater shift than interventions targeting a narrow range of products?)
    - Direction (i.e. do individuals react to price increases and decreases in an equivalent fashion or does the direction of the price change have an independent effect?)
    - Information (i.e. does the method by which individuals are informed about the price change result in different responses?)
- How will changes in food price affect the total price of the diet for different groups defined by socioeconomic or other personal characteristics?

Search strategy

PubMed, EMBASE, Web of Science, PsychInfo, and EconLit will be searched to identify potentially relevant studies. The search strategy for PubMed is as follows, with equivalent searches conducted in the other databases:

1. food OR foods OR snack OR snacks OR beverage* OR “soft drink” OR soda OR “carbonated drink”
2. fruit* OR vegetable* OR cereal* OR candy OR sweets OR confectionary OR chocolate* OR meat OR dairy
3. sugar OR sugars OR sugary OR “energy dense” OR “energy density” OR fat OR fats OR saturates OR “saturated fat” OR salt OR sodium OR fibre OR fiber
4. 1 OR 2 OR 3
5. tax OR taxation OR taxes OR taxed OR subsidy OR subsidies OR price OR prices OR discount OR discounts

[a] Personal characteristics rather than individual characteristics will be the focus due to the expectation that included studies may look at households’ rather than individuals’ purchases

6. experiment OR experimental OR trial OR test OR supermarket* OR shop OR shops OR store OR stores OR controlled OR participant* OR intervention OR interventions OR random OR randomised OR randomized
7. 4 AND 5 AND 6

In addition to the above, the reference lists of included papers and relevant reviews[7,10,11] will be hand searched to identify any additional articles of interest. The title and abstract of identified studies will be screened for the inclusion criteria and full text versions of relevant articles will be retrieved.

A single researcher will complete the screening process with a second researcher cross-checking a 10% sample of excluded titles for relevance.

Inclusion criteria

An article will be included if it:

- is a controlled experimental study[b]
- reports results stratified by the socioeconomic or other personal characteristics[c] of study participants. These could be secondary analyses, and the study need not be adequately powered to examine differences by personal characteristics.
- examines the effect of price change[d] on food or beverage purchases
- collects individual level[e] data to determine the effect (i.e. excluding single site studies such as vending or cafeteria studies where the outcome is sales of targeted food)
- has one of the following outcome measures:
    - price elasticity
    - changes in purchases/consumption of targeted or non-targeted foods

An article will be excluded if it:

- examines price changes of alcoholic drinks in isolation
- is a review or commentary article where no original data are presented

[b] Both studies with an independent control group and studies which use individuals' previous purchases as the control will be included. The study setting may include studies of real-life price changes and choice experiments where real-life purchasing behaviour is not measured.

[c] Personal characteristics refer to both modifiable and unmodifiable individual level features that may plausibly influence the response of an individual to a change in food/beverage prices. This includes factors such as age, sex, personality measurements (e.g. impulsivity as measured by the stop-signal task), socio-economic status, income and education.

[d] Experimental studies may phrase the price changes applied as a tax/subsidy or may refer simply to the value of the price change. In this review, we will not discriminate on studies based on the description of the pricing intervention, provided that a price intervention is applied independently of other interventions

[e] Experimental studies focusing on food price are concerned with food purchasing behavior, and food purchasing is often conducted at the household-level. Therefore, the use of 'individual' in this protocol includes household-level studies, but does not include studies where total volumes of sales of specific food products are the units of analysis.

- does not test pricing interventions in isolation from other interventions (e.g. tailored nutrition education)[f]
- examines exclusively children's (<18 years of age) purchases
- was published prior to January 1980
- full text not available in English

Analysis

Due to the anticipated heterogeneity in price changes tested, experimental settings, and populations investigated, no meta-analysis of the data will be conducted. However, results will be collated to allow for comparison across similar study types and outcomes, complemented by a data extraction table to summarize relevant information from the extracted studies. The full data extraction table will be collated in Microsoft Excel.

The first table presented in the report will be a summary table to provide the reader with an overview of the characteristics of the studies included in the review. The following data will be presented:

- Author name (year)
- Country
- Setting
- Study design
- Intervention(s)
- Personal characteristic(s) examined
- Analysis method

Where possible, the results from included studies will be reported as the price elasticity for the targeted food(s), by personal characteristic. Studies will be grouped according to the type of personal characteristic that is analysed: non-modifiable (age, sex), individual (personality (e.g. impulsivity, restraint), BMI, hunger) and societal (income, socio-economic status, education). Results from indirect measures (e.g. impact of budget where budget is determined by a combination of income and household size) will not be reported. Where applicable, the results of fully adjusted models will be the results reported in the data extraction table. In situations where an effect by a personal characteristic is reported but complete data are not given, study authors will be contacted and asked to provide the numeric results.

Following a description of the characteristics of the included studies, the following table will be presented in order to synthesise the information relevant to the primary research question of the review. The table has been populated with some example studies to demonstrate layout and reporting. An effort will be made to contact the authors where price elasticity estimates cannot be

[f] Where the price change is accompanied by information to participants about the extent of a price change, the study will be included. However, where an additional behavioural intervention is implemented, or where the information given to participants goes beyond mere information about the magnitude of the price (e.g. if price change reduction label was applied in combination with a health message), the article will be excluded from this review

derived from the article; numerical results will be reported as given in the paper where it remains impossible to determine price elasticities.

Personal characteristic	Personal characteristic measure	Author name (year)	Target food	Price elasticity result (95% confidence intervals)
Non-modifiable<br>- Sex	a) Male<br>b) Female	Example (2014)	Fruit	a) -1.0 (-0.8, -1.2)<br>b) -0.6 (-0.7, -0.5)
		Example (2014)	High-calorie foods	a) -1.2 (-1.0, -1.4)<br>b) -0.6 (-0.7, -0.5)
Modifiable<br>- Income	a) >NZ$60,000<br>b) <NZ$60,000	Example (2014)	Fruit and vegetables	a) -1.0 (-0.8, -1.2)<br>b) -0.6 (-0.7, -0.5)
	a) £0-£10k<br>b) £10k-£20k<br>c) £20k-£50k<br>d)>£50k	Example (2014)	Fruit and vegetables	a) -1.0 (-0.8, -1.2)<br>b) -0.6 (-0.7, -0.5)<br>c) -1.2 (-1.0, -1.4)<br>d) -0.6 (-0.7, -0.5)

In addition, to give an overview of the types of study that have been conducted and the extent to which the review will be able to address the secondary research questions, a count of the number of studies according to the following characteristics will be presented:

- Does the study give the differences in purchases of non-targeted products?
- Were substitution effects examined? (Yes/No)
- Did the study look at multiple price conditions for the target product(s)? (Yes/No)
- Did the study examine both increases and decreases for the target product? (Yes/No)
- Did the study examine more than one target product/product group? (Yes/No)
- Were individuals informed about the price change and how? (Yes (methods)/No)
- Did the study examine change in the total diet cost?

If there are no studies examining a secondary research question, then it will be noted that further research is needed to build the evidence base in this area. If there are between one and three studies addressing a secondary research question, the evidence will be summarized narratively. If there are more than three studies addressing a particular research question, then relevant data will be tabulated in addition to a narrative commentary so as to provide an accessible summary for the reader.

Study quality

Study quality will be assessed according to the Cochrane Risk of Bias tool[12]. The following criteria will be used to present study quality (in relation to the primary research question) for all included studies and will be summarised in the report of the systematic review.

Bias domain	Relevant questions	Scoring	Support for judgement
Selection bias	Were participants randomised to the study [price] condition?<br><br>Were participants representative of the target population?<br><br>Were participant recruitment methods independent of personal characteristics?	Low risk if Yes<br>High Risk if No<br>Unclear if no information given	Quote from included study where available, comment where not.
Performance bias	Were participants blinded to the aims of the research study (i.e. blinded to price changes)?<br><br>Did the study design require participants to make actual purchases using their own money?		
Detection bias	Were participants blinded to the outcome of interest (i.e. the reasons why prices had changed)?<br><br>Were researchers blinded to the allocation of participants?		
Attrition bias	Was complete outcome data obtained?<br><br>Was attrition unrelated to the personal characteristics examined?		
Reporting bias	Did the study set out to look at differences by personal characteristics?		

In addition, a narrative sensitivity analysis will be conducted to determine the extent to which the conclusions drawn are dependent on whether the study aimed to investigate differential responses by personality characteristic(s). This narrative sensitivity analysis will be conducted in lieu of a numeric sensitivity analysis or funnel plot; this is due to the anticipated heterogeneity of included studies and the anticipated inability to determine whether included studies were sufficiently powered to detect differences in responses by personal characteristics.

Conclusion

Increasing the price of unhealthy foods and decreasing the price of healthy foods has been suggested as one method by which to improve population diets. Prior to the implementation of such policies, an understanding of the anticipated effect of such policies is required. This includes an understanding of how personal characteristics may moderate the effectiveness of fiscal measures to improve population diets. Experimental studies are able to provide evidence as to the potential effect of price changes on purchases, diets, and health outcomes. This systematic review aims to synthesise the evidence from experimental studies in order to determine how individuals respond to food/beverage price changes in experimental settings. It differs from a previous review of experimental studies[11] in the systematic approach adopted and the precise research questions of interest.

References

1. World Health Organisation. *Global health risks: Mortality and burden of disease attributable to selected major risks*. (2009).

2. Andreyeva, T., Long, M. W. & Brownell, K. D. The impact of food prices on consumption: a systematic review of research on the price elasticity of demand for food. *Am. J. Public Health* **100,** 216–22 (2010).

3. Rao, M., Afshin, A., Singh, G. & Mozaffarian, D. Do healthier foods and diet patterns cost more than less healthy options? A systematic review and meta-analysis. *BMJ Open* **3,** e004277 (2013).

4. Mittal, B. Integrated Consumer Coupon Framework for Relating Diverse Characteristics to Supermarket Redemption. *J. Mark.* **31,** 533–544 (1994).

5. Raghubir, P. Coupon Value : Signal for Price ? *J. Mark. Res.* **35,** 316–324 (1998).

6. Green, R. *et al.* The effect of rising food prices on food consumption: systematic review with meta-regression. *BMJ* **346,** f3703 (2013).

7. Thow, A. M., Jan, S., Leeder, S. & Swinburn, B. The effect of fiscal policy on diet, obesity and chronic disease: a systematic review. *Bull. World Health Organ.* **88,** 609–14 (2010).

8. Nederkoorn, C., Havermans, R. C., Giesen, J. C. A. H. & Jansen, A. High tax on high energy dense foods and its effects on the purchase of calories in a supermarket. An experiment. *Appetite* **56,** 760–5 (2011).

9. Eyles, H., Ni Mhurchu, C., Nghiem, N. & Blakely, T. Food pricing strategies, population diets, and non-communicable disease: a systematic review of simulation studies. *PLoS Med.* **9,** e1001353 (2012).

10. An, R. Effectiveness of subsidies in promoting healthy food purchases and consumption: a review of field experiments. *Public Health Nutr.* **16,** 1215–28 (2013).

11. Epstein, L. H. *et al.* Experimental research on the relation between food price changes and food-purchasing patterns: a targeted review. *Am. J. Clin. Nutr.* **95,** 789–809 (2012).

12. Higgins, J. P. T. *et al.* The Cochrane Collaboration's tool for assessing risk of bias in randomised trials. *BMJ* **343,** d5928 (2011).
